# Effect of *Echinacea purpurea* extract and powder with reduced sodium nitrite on quality, oxidative stability, and microbial safety of dry-fermented duck sausage

**DOI:** 10.1016/j.fochx.2025.102774

**Published:** 2025-07-10

**Authors:** Osama I.A. Soltan, Hanaa S.S. Gazwi, Galiya R. Yusupova, Andrey P. Gerasimov, Awatif M. Almehmadi, Reem M.E. Magdy

**Affiliations:** a Department of Food Science, Faculty of Agriculture, Minia University, Minia 61519, Egypt; b Department of Agricultural Chemistry, Faculty of Agriculture, Minia University, El-Minia 61519, Egypt; c Kazan State Academy of Veterinary Medicine named after N.E. Bauman, 420029, 35 Sibirsky Trakt St., Kazan, Russia; d Kazan National Research Technological University, 420015, 68 Karl Marx st., Kazan, Russia; e Department of Clinical Nutrition, Faculty of Applied Medical Sciences, Umm AL-Qura University, P.O. BOX. 715, Makkah 21955, Saudi Arabia

**Keywords:** *Echinacea purpurea*, Fermentation, Lipid oxidation

## Abstract

This study investigated the impact of incorporating *Echinacea purpurea* extract and powder, alongside a 50 % reduction in sodium nitrite, on the quality of dry-fermented duck sausages. Treatments involved 0.05 %, 0.1 %, and 0.2 % concentrations of *E. purpurea* in either extract or powder form. All treated sausages maintained high protein levels, while fat and ash contents varied. Moisture decreased during ripening, resulting in weight loss without significant differences between treated and control groups. The pH declined during fermentation and slightly increased during maturation. Total acidity ranged from 0.54 % to 0.69 %. The addition of *E. purpurea* significantly lowered thiobarbituric acid and peroxide values, indicating improved oxidative stability. Color analysis revealed enhanced redness (a*) and acceptable lightness (L*), with some treatments performing comparably to the nitrite-only control. Microbiological analysis confirmed the absence of pathogenic bacteria. Overall, incorporating *E. purpurea* improved the microbiological safety, physicochemical quality, and oxidative stability of fermented duck sausages, even with reduced sodium nitrite levels.

## Highlights

• Echinacea improved the oxidative stability of fermented duck sausages.
• Reduced nitrite with Echinacea maintained product quality and safety.
• Echinacea extract and powder showed strong antimicrobial properties.
• Color and texture were enhanced by adding Echinacea to formulations.
• Echinacea is a promising natural alternative to synthetic preservative.

## 1 Introduction

Dry-fermented sausages (DFS) are high-value cured meat products known for their distinct sensory properties and potential health benefits (Pugliese & Sirtori, 2012). Their formulation typically includes minced meat and fat, combined with salt, spices, sugar, starter cultures, and various functional additives such as dietary fibers and vitamins to enhance both nutritional and technological qualities (Nikolić et al., 2020).

Despite their popularity, DFS are particularly vulnerable to lipid oxidation and microbial spoilage due to their high fat content (15–40 %), lack of thermal treatment, and variability in raw material quality. These factors present ongoing challenges to product stability and consumer safety (Tomović et al., 2020).

To improve shelf life and ensure microbiological safety, several additives are used in DFS processing. Among them, sodium nitrite is widely applied for its well-established antioxidative and antimicrobial properties (Ozaki et al., 2020; Zhu et al., 2020). In cured meats, nitrite plays multiple roles: it enhances flavor, stabilizes color and aroma, and inhibits lipid oxidation. Its reaction with myoglobin leads to the formation of nitrosyl myoglobin, which imparts the characteristic cured red color (Parthasarathy & Bryan, 2012; Škrlep et al., 2022).

Crucially, nitrite also inhibits pathogens such as *Clostridium botulinum* by interfering with iron‑sulfur enzymes essential for bacterial energy metabolism (Ford & Lorkovic, 2002). However, growing health concerns have been raised regarding the excessive intake of nitrites, which may lead to the formation of carcinogenic nitrosamines and other adverse cardiovascular effects (Milkowski et al., 2010).

In response, recent research has focused on plant-based alternatives that can mimic the preservative functions of nitrites. Numerous studies have investigated the use of extracts from herbs, fruits, and agro-industrial by-products, many of which are rich in polyphenols with strong antioxidant and antimicrobial properties (Sharma et al., 2021; Tian et al., 2020).

One promising candidate is *Echinacea purpurea*, a medicinal plant traditionally used in Europe, Australia, and North America for its immunomodulatory and therapeutic effects (Speroni et al., 2002). It contains a variety of bioactive compounds such as caftaric acid, chlorogenic acid, cichoric acid, echinacoside, and other phenolic derivatives (Merati & Farshad, 2020; Oniszczuk et al., 2019). Recent interest has focused on its potential role in meat products, not only for improving quality but also for contributing functional health benefits (Difonzo et al., 2022).

This study investigates the use of *E. purpurea* extract and powder as natural additives in fermented duck sausage. It aims to assess their impact on microbiological safety, physicochemical quality, and shelf life, while serving as partial replacements for sodium nitrite. This approach supports the development of cleaner-label products with reduced health risks associated with synthetic preservatives.

## 2 Material and methods

### 2.1 Raw materials

Duck breast fillets with skin and duck fat were obtained from the Agricultural Supply and Marketing Consumer Cooperative “Kausar,” located at Adolf-Schemel-Str. 9, Salzburg, Austria. Curing salt (containing 0.6 % NaNO₂), food-grade salt, Echinacea extract or powder, sugar, black pepper powder, dried garlic powder, nutmeg powder, and starter cultures (“Biobak P" containing *Lactobacillus sakei* and *Staphylococcus xylosus*) were also sourced from Wiberg, Adolf-Schemel-Str. 9, Salzburg, Austria.The equipment used included a mincer (PAL-130, Fatosa, Barcelona, Spain), a paddle mixer (REVIK RX-200, Poland), ICell Premium casings (Atlantis-Pak Co., Russia), a vacuum sausage filler (Rex VF 327, REX Technologie GmbH & Co. KG), and sausage frames installed in a REICH AIRMASTER KKRI 100005 unit (REICH Co., Germany). All reagents and chemicals used in the study were of analytical grade and purchased from Fisher Scientific, UK. The entire study was conducted in a meat processing facility operated by the Agricultural Supply and Marketing Consumer Cooperative “Kausar” in Kazan, Republic of Tatarstan, Russia.

### 2.2 Preparation of *E. purpurea* extract and powder

Whole *E. purpurea* herb was ground into a fine powder. The ethanolic extract was prepared by macerating the powder in 70 % ethanol (1:10 *w*/*v*) with magnetic stirring for 3 h at room temperature. The mixture was then filtered through Whatman filter paper, and the solvent was evaporated at 50 °C using a rotary evaporator. The resulting dry extract and powder were stored at −18 °C in vacuum-sealed bags until further use in the preparation of dry fermented sausages.

The phytochemical constituents of the ethanolic extract of *E. purpurea* were analyzed using gas chromatography–mass spectrometry (GC/MS) to identify the major bioactive compounds responsible for its functional properties. GC/MS analysis was performed using a Thermo Scientific TG-5MS capillary column (30 m × 0.25 mm, 0.1 μm film thickness). The system operated with electron ionization at 70 eV, using helium as the carrier gas at a flow rate of 1 mL/min. The injector and MS transfer line temperatures were set at 280 °C. Compounds were identified by comparing their retention times and mass spectra with those in the NIST and WILLY libraries (Adams, 2007), and quantified based on their relative peak areas.

### 2.3 Preparation of DFS

The preparation steps for DFS are illustrated in Fig. S1, while the formulation is presented in Table S1. The ingredients used for the DFS were prepared based on the method described by Stajic, Perunovic, Stanisic, Žujovic, & Živovic, 2012, with slight modifications.

### 2.4 Physiochemical analyses of DFS

The samples from each treatment were analyzed for moisture, protein, fat, and ash content following the methods described by AOAC (2006). Total carbohydrate content was calculated by difference.

### 2.5 Determination of pH and total acidity

The pH of the samples was measured using a digital pH meter (Ramadhan et al., 2011). Total acidity was determined according to the method described by Friedrich (2001).

### 2.6 Weight loss (%)

The weight loss (%) was determined according to equation:

$$\text{Weight loss}\;\left(\%\right)=\frac{W_{0}-W_{d}}{W_{0}}\times 100$$

where: W_d_ = weight of the product on the sampling day during processing (g);

W_0_ = weight on day 0 of the processing (g).

### 2.7 Determination of color (L, a, and b) and ∆E

Color values (L, a, b) of fermented duck sausage were measured using a Color Tec PCM Color Meter (NJ, USA). L indicates lightness, a represents redness, and b shows the blue-to-yellow chromatic range (Stajic, Perunovic, Stanisic, Žujovic, & Živovic, 2012). Additionally, ∆E was calculated to assess color differences between the control (C2) and other samples.

$$∆E={\left({\left(L_{\text{C1}}–L_{N}\right)}^{2}+{\left(a_{\text{C1}}–a_{N}\right)}^{2}+\left(b_{\text{C1}}–{b_{N}}^{2}\right)\right)}^{½}$$

### 2.8 Determination of peroxide value

One gram of fat from each sausage sample was mixed with 15 mL of acetic acid–chloroform solution (3:2) in a conical flask. After adding 1 mL of saturated potassium iodide and 20 mL of distilled water, the mixture was titrated with 0.01 N sodium thiosulfate using 1 % starch as an indicator. A blank was prepared similarly without fat. PV was calculated and expressed as milliequivalents of peroxide per kg of sample.

$$\mathrm{PV}\;\left(\mathrm{meq}/\mathrm{kg}\right)=\frac{\left(S\right)\times N}{\mathrm{Weight of Sample}\;\left(g\right)}\times 100$$

where: N is the Normality of sodium thiosulfate (0.01 N), S = Volume of titration.

### 2.9 Determination of Thiobarbituric acid

Thiobarbituric acid (TBA) was assessed using the distillation method per AOCS (2006). Absorbance was measured with a T80 UV/Vis spectrophotometer (PG Instruments LTD).

### 2.10 Microbiological analysis:

Microbial counts for total *coliforms*, *Staphylococcus aureus*, *Listeria monocytogenes*, *Salmonell*a, *E. coli*, and *Clostridium* were conducted following AOAC (2006), ISO, n.d. 6888-3:2003, ISO, n.d. 11290-2:2017, ISO, n.d. 6579:2002, and ISO, n.d.7218:2007 standards.

### 2.11 Sensory evaluation

Sensory attributes—including taste, odor, color, texture, and overall acceptability—were evaluated according to the method of Fernández-López et al. (2019). A panel of untrained assessors evaluated the sensory quality of dry-fermented sausage samples using a validated questionnaire.

### 2.12 Statistical analysis

Data were analyzed using SPSS version 24 (SPSS Inc., Chicago, IL, USA). All tests were performed in triplicate, and results were reported as mean ± standard deviation. Group means were compared using Duncan's multiple range test.

## 3 Results and discussion

### 3.1 Identification of bioactive compounds in *E. purpurea* ethanolic extract using GC/MS

As shown in Table S2 and Fig. S2, twenty-nine major compounds were identified in the *E. purpurea* extract. The most abundant components were 9,12-octadecadienoic acid (Z,Z) (22.14 %), n-hexadecanoic acid (14.79 %), cis-5,8,11,14,17-eicosapentaenoic acid (5.53 %), 4H-pyran-4-one, 2,3-dihydro-3,5-dihydroxy-6-methyl (4.84 %), and 9-octadecenoic acid (Z) (4.29 %). These bioactive constituents are known for their antioxidant and antimicrobial properties, which support the potential use of *E. purpurea* extract as a natural preservative in meat products.

Synthetic nitrites are widely used in meat processing due to their proven efficacy in microbial inhibition, lipid oxidation prevention, and color stabilization (Koç, 2017). However, under certain conditions, they may interact with amines to form N-nitrosamines compounds associated with carcinogenic risk (Li et al., 2013). In light of these health concerns, this study explores the potential of *E. purpurea* extract and powder as natural alternatives to partially replace sodium nitrite in dry-fermented duck sausages, aiming to enhance safety while maintaining product quality.

### 3.2 Physicochemical of DFS

**Fig. 1:**
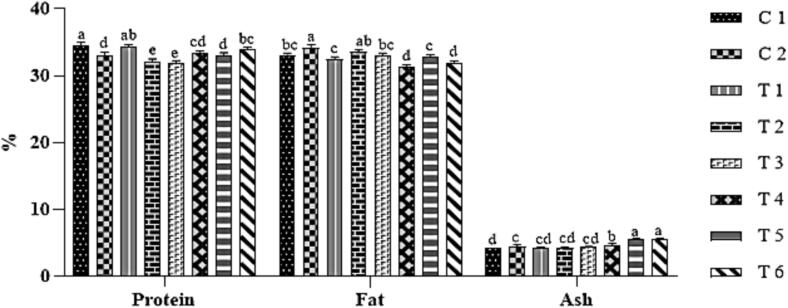
Chemical composition of fermented sausage during the preparation process: C1: Negative control with no curing salt or *E. purpurea* (0 mg/kg NaNO₂). C2: Positive control containing 1.35 % curing salt (equivalent to 81 mg NaNO₂/kg meat). T1–T3: Treated with 0.05 %, 0.1 %, and 0.2 % *E. purpurea* extract, respectively, each combined with 0.67 % curing salt (equivalent to 40.2 mg NaNO₂/kg meat). T4–T6: Treated with 0.05 %, 0.1 %, and 0.2 % *E. purpurea* powder, respectively, each combined with 0.67 % curing salt (equivalent to 40.2 mg NaNO₂/kg meat).

The protein, fat, and ash contents of the dry-fermented sausage (DFS) samples are shown in Fig. 1. All samples exhibited high protein levels, ranging from 31.8 % to 33.8 %, consistent with the known nutritional profile of duck meat, which is characterized by higher fat content compared to other poultry meats. Statistical analysis revealed significant differences in fat content among treatments (*p* ≤ 0.05). This aligns with the findings of Kim et al. (2017), who reported increased fat content in duck ham with the inclusion of duck skin.

The highest ash content was recorded in treatments T5 and T6, while the control group (C1) showed the lowest values. The addition of 0.1 % and 0.2 % *E. purpurea* powder significantly increased ash levels. These results are in agreement with Ozaki et al. (2021), who observed elevated ash content in fermented sausages supplemented with 0.5 % vegetable powders.

**Fig. 2:**
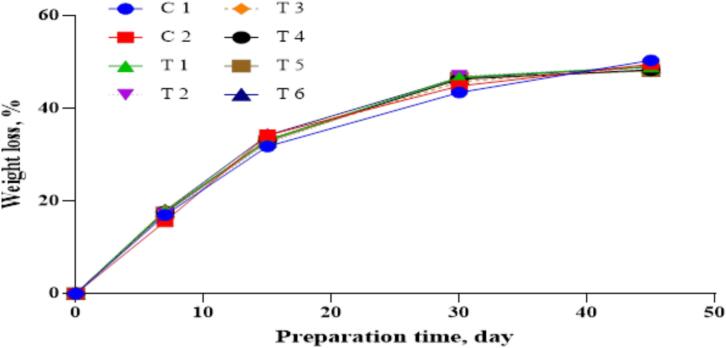
Weight loss (%) of dry-fermented sausage samples during the preparation process. C1: Negative control with no curing salt or *E. purpurea* (0 mg/kg NaNO₂). C2: Positive control containing 1.35 % curing salt (equivalent to 81 mg NaNO₂/kg meat). T1–T3: Treated with 0.05 %, 0.1 %, and 0.2 % *E. purpurea* extract, respectively, each combined with 0.67 % curing salt (equivalent to 40.2 mg NaNO₂/kg meat). T4–T6: Treated with 0.05 %, 0.1 %, and 0.2 % *E. purpurea* powder, respectively, each combined with 0.67 % curing salt (equivalent to 40.2 mg NaNO₂/kg meat).

**Fig. 3:**
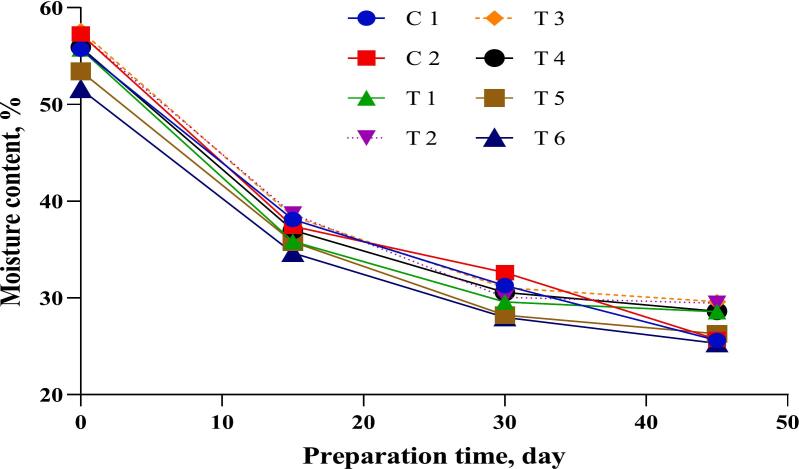
Moisture content (%) of dry-fermented sausage samples during the preparation process. C1: Negative control with no curing salt or *E. purpurea* (0 mg/kg NaNO₂). C2: Positive control containing 1.35 % curing salt (equivalent to 81 mg NaNO₂/kg meat). T1–T3: Treated with 0.05 %, 0.1 %, and 0.2 % *E. purpurea* extract, respectively, each combined with 0.67 % curing salt (equivalent to 40.2 mg NaNO₂/kg meat). T4–T6: Treated with 0.05 %, 0.1 %, and 0.2 % *E. purpurea* powder, respectively, each combined with 0.67 % curing salt (equivalent to 40.2 mg NaNO₂/kg meat).

Fig. 2, Fig. 3 present the changes in moisture content and weight of DFS samples during processing. A consistent decline in moisture was observed in all groups, primarily due to water evaporation, resulting in a proportional decrease in product weight. These trends are comparable to those reported by Carvalho et al. (2017). However, the total weight loss in the current study was lower than that reported by Ozaki et al. (2021), who documented losses ranging from 41.46 % to 42.74 % in sausages enriched with vegetable powders after fermentation.

Weight loss during fermentation is affected by environmental factors such as processing time, temperature, and relative humidity. Rocchetti et al., (2020) emphasized the role of humidity in controlling moisture evaporation and weight reduction. In the present study, no significant differences were observed between control and treated groups in terms of weight loss at the end of fermentation. Treatments T2 and T3, containing *E. purpurea* extract, retained the highest moisture content by the end of ripening. These moisture values closely corresponded with the observed weight loss percentages, as shown in Fig. 2, Fig. 3.

### 3.3 Changes in pH and Total acidity during fermentation

**Fig. 4:**
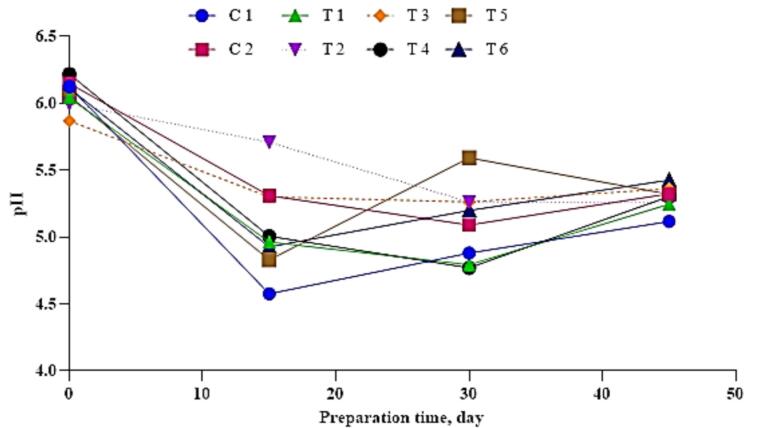
**Changes in pH values during the fermentation and maturation of duck sausage treated with varying levels of Echinacea extract and powder.** C1: Negative control with no curing salt or *E. purpurea* (0 mg/kg NaNO₂). C2: Positive control containing 1.35 % curing salt (equivalent to 81 mg NaNO₂/kg meat). T1–T3: Treated with 0.05 %, 0.1 %, and 0.2 % *E. purpurea* extract, respectively, each combined with 0.67 % curing salt (equivalent to 40.2 mg NaNO₂/kg meat). T4–T6: Treated with 0.05 %, 0.1 %, and 0.2 % *E. purpurea* powder, respectively, each combined with 0.67 % curing salt (equivalent to 40.2 mg NaNO₂/kg meat).

Fig. 4 and Table 1 present the evolution of pH and total acidity (TA) during the fermentation and maturation of the sausages. A general decline in pH was observed across all treatments during fermentation, primarily due to lactic acid production by lactic acid bacteria (LAB), in agreement with findings by Ramírez et al. Ramirez et al. (2022). The use of commercial starter cultures enhanced acidification and inhibited the growth of undesirable microorganisms. A slight increase in pH at the end of maturation was also noted, likely due to the proteolytic release of alkaline compounds, as reported by Gomez Lorenzo (2013) and Ozaki et al. (2021).

**Table 1:** Evolution of TA (% lactic acid) in Fermented Sausage Throughout the Preparation Process.

	**Zero time**	**15 days**	**30 days**	**45 days**
**C1**	0.45 c ± 0.01	0.80 a ± 0.11	0.78 a ± 0.06	0.64 a ± 0.01
**C2**	0.46 c ± 0.12	0.64 c ± 0.02	0.61 cd ±0.03	0.54 b ± 0.11
**T1**	0.46 c ± 0.01	0.59 de ±0.02	0.65 bc ±0.07	0.67 a ± 0.07
**T2**	0.45 c ± 0.11	0.55 e ± 0.12	0.67 bc ±0.04	0.55 b ± 0.02
**T3**	0.52 a ± 0.02	0.61 cd ±0.03	0.56 d ± 0.03	0.55 b ± 0.15
**T4**	0.46 c ± 0.01	0.75 b ± 0.06	0.66 bc ±0.12	0.65 a ± 0.05
**T5**	0.45 c ± 0.05	0.47 f ± 0.02	0.70 b ± 0.11	0.69 a ± 0.08
**T6**	0.50 b ± 0.15	0.83 a ± 0.13	0.64 bc ±0.02	0.57 b ± 0.03

Values are expressed as mean ± SD (*n* = 3). Means within the same column not sharing the same superscript letter (a–c) are significantly different at *P* ≤ 0.05. C1: Negative control with no curing salt or *E. purpurea* (0 mg/kg NaNO₂).C2: Positive control containing 1.35 % curing salt (equivalent to 81 mg NaNO₂/kg meat). T1–T3: Treated with 0.05 %, 0.1 %, and 0.2 % *E. purpurea* extract, respectively, each combined with 0.67 % curing salt (equivalent to 40.2 mg NaNO₂/kg meat). T4–T6: Treated with 0.05 %, 0.1 %, and 0.2 % *E. purpurea* powder, respectively, each combined with 0.67 % curing salt (equivalent to 40.2 mg NaNO₂/kg meat).

The lowest final pH value was recorded in the control group (C1) (*p* < 0.05), while T1 and T3 reached 5.24 and 5.36, respectively values consistent with those reported by Natalia et al. (2019). Final pH was influenced by multiple factors, including the type of starter culture, fermentation temperature, and sugar content.

Total acidity exhibited an inverse pattern relative to pH, with higher acidity associated with lower pH values. At the beginning of fermentation, T3 had a TA of 0.52 %, which increased to 0.64 % in the C1 group by the end of ripening. Overall, TA values ranged from 0.54 % to 0.69 %, consistent with the findings of Serdaroglu et al. Serdaroglu et al. (2023).

### 3.4 Thiobarbituric acid (TBA) values

Oxidation is a major factor in the deterioration of meat products during storage and air-drying, especially in lipid-rich items such as dry sausages. It negatively impacts organoleptic and nutritional quality by causing off-flavors, discoloration, and texture degradation, primarily through the oxidation of unsaturated fatty acids during processing steps like mincing, stuffing, and drying (Cunha et al., 2018; Lorenzo et al., 2018). Lipid peroxidation also leads to the loss of essential nutrients and the formation of harmful secondary products such as malondialdehyde (MDA), a rancid-smelling compound commonly measured using the thiobarbituric acid (TBA) assay.

**Fig. 5:**
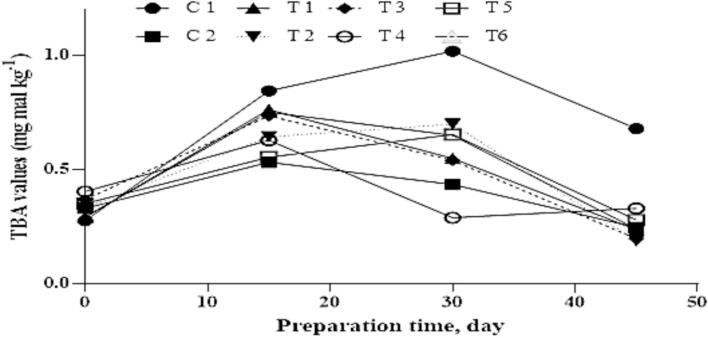
**TBA values (expressed as mg MDA kg**^**−1**^**sample) during the preparation process of dry fermented sausage.** C1: Negative control with no curing salt or *E. purpurea* (0 mg/kg NaNO₂). C2: Positive control containing 1.35 % curing salt (equivalent to 81 mg NaNO₂/kg meat). T1–T3: Treated with 0.05 %, 0.1 %, and 0.2 % *E. purpurea* extract, respectively, each combined with 0.67 % curing salt (equivalent to 40.2 mg NaNO₂/kg meat). T4–T6: Treated with 0.05 %, 0.1 %, and 0.2 % *E. purpurea* powder, respectively, each combined with 0.67 % curing salt (equivalent to 40.2 mg NaNO₂/kg meat).

As shown in Fig. 5, TBA values increased in all samples after day 0, indicating progressive lipid oxidation. The control group (C1), which contained neither sodium nitrite nor *E. purpurea*, exhibited the highest TBA levels throughout ripening, underscoring the importance of antioxidant protection. These results are consistent with Ozaki et al. (2021), who reported elevated TBA values in nitrite-free fermented meats.

Treatments T2 and T3, which included 0.1 % and 0.2 % *E. purpurea* extract along with reduced sodium nitrite, showed significantly lower TBA values by day 45, likely due to the antioxidant properties of the plant (Zuparova & Ismoilova, 2022; Juki et al., 2015). Interestingly, TBA values began to decline in all treatments after 30 days, possibly due to interactions between lipid oxidation products and myofibrillar proteins during fermentation, as proposed by Xiong et al. (2015).

These findings support the use of natural antioxidants as viable alternatives to synthetic nitrites (Lorenzo et al., 2018). Treatments T1–T3 effectively preserved color and reduced MDA formation, with T2 and T3 offering enhanced oxidative stability and acceptable sensory characteristics. Given the safety concerns associated with the full nitrite dose in C2, these treatments provide safer and more consumer-friendly options.

### 3.5 Peroxide value

**Fig. 6:**
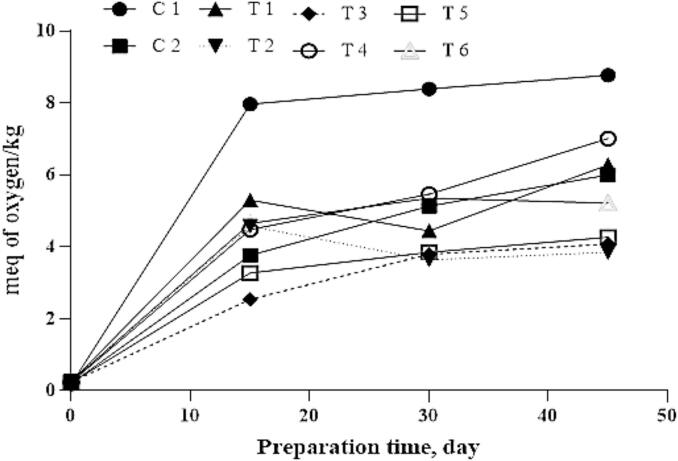
**Peroxide value of fermented sausage.** C1: Negative control with no curing salt or *E. purpurea* (0 mg/kg NaNO₂).C2: Positive control containing 1.35 % curing salt (equivalent to 81 mg NaNO₂/kg meat). T1–T3: Treated with 0.05 %, 0.1 %, and 0.2 % *E. purpurea* extract, respectively, each combined with 0.67 % curing salt (equivalent to 40.2 mg NaNO₂/kg meat). T4–T6: Treated with 0.05 %, 0.1 %, and 0.2 % *E. purpurea* powder, respectively, each combined with 0.67 % curing salt (equivalent to 40.2 mg NaNO₂/kg meat).

Peroxide value (PV) is a key indicator of primary lipid oxidation, reflecting the accumulation of hydroperoxides. Due to its high fat content, duck meat is particularly susceptible to oxidative deterioration, which can adversely affect flavor and aroma. As shown in Fig. 6, significant differences (*p* < 0.05) in PV were observed among treatments during the 45-day ripening period.

All samples exhibited increased PVs over time, indicating progressive lipid oxidation. However, the control group (C1) recorded the highest PV by the end of ripening, suggesting more advanced oxidative degradation in the absence of protective agents. In contrast, treatments T2, T3, and T5 supplemented with *E. purpurea* showed significantly lower PVs, indicating a reduced formation of hydroperoxides. This reduction is likely attributable to the antioxidant capacity of Echinacea compounds, which appear to mitigate lipid peroxidation during fermentation and storage.

These findings highlight the potential of *E. purpurea* to enhance oxidative stability in dry-fermented sausages by suppressing hydroperoxide formation. The results are in agreement with Aarland et al. (2017), who reported notable antioxidant effects of Echinacea extracts in food systems.

### 3.6 Color properties of the fermented sausage

Fig. S3 illustrates the changes in color parameters across all treatment groups throughout the sausage production process. As noted by Fonseca et al. (2015), color development in fermented sausages is influenced by both fermentation and drying stages.

At day 0, L* values (lightness) were significantly higher in groups C1, C2, and T1. The elevated lightness observed in C1, which lacked both nitrate and nitrite, can be attributed to the absence of nitrosylmyoglobin formation. This trend aligns with Serdaroglu et al. Serdaroglu et al. (2023), who reported increased lightness in nitrite-free sausages during ripening.

As fermentation progressed, L* values gradually declined in all samples, likely due to pigment formation and moisture loss, consistent with findings by Ozaki et al. (2021). The nitrite-treated group (C2) developed a characteristic bright red color resulting from nitrosylmyoglobin formation, as also reported by Ferysiuk and Wójciak (2020).

Regarding a* values (redness), group T2 containing 0.1 % *E.purpurea* extract showed a significant increase by day 45, indicating enhanced red color intensity. This observation supports the findings of Ali et al. (2007), who reported higher a* and lower L* values in duck meat compared to chicken. Notably, the redness in Echinacea-treated groups was comparable to that of the nitrite control (C2), suggesting that *E. purpurea* may serve as a natural color enhancer in fermented sausages.

In contrast, b* values (yellowness) decreased steadily during ripening. By the end of fermentation, C1 displayed the lowest b* values among all treatments.

**Fig. 7:**
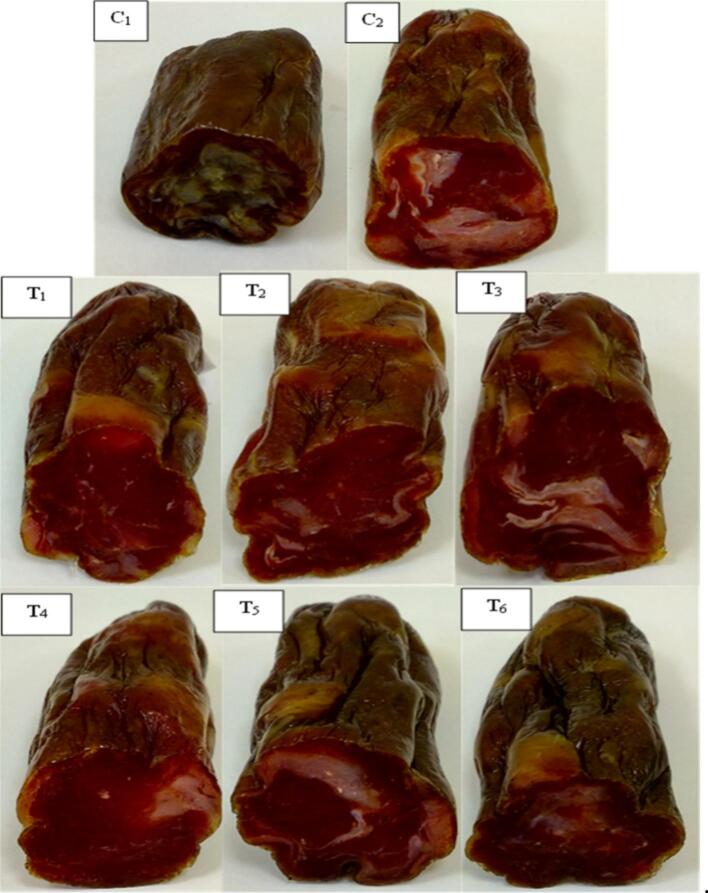
**Changes in color of fermented sausage** after **preparation**C1: Negative control with no curing salt or *E. purpurea* (0 mg/kg NaNO₂). C2: Positive control containing 1.35 % curing salt (equivalent to 81 mg NaNO₂/kg meat). T1–T3: Treated with 0.05 %, 0.1 %, and 0.2 % *E. purpurea* extract, respectively, each combined with 0.67 % curing salt (equivalent to 40.2 mg NaNO₂/kg meat). T4–T6: Treated with 0.05 %, 0.1 %, and 0.2 % *E. purpurea* powder, respectively, each combined with 0.67 % curing salt (equivalent to 40.2 mg NaNO₂/kg meat).

The total color difference (ΔE) was calculated to assess visual differences between the nitrite control (C2) and the other groups. ΔE values exceeding 2.0 indicate perceptible color differences (Serdaroglu et al., 2023). In this study, all treatments except T1 and T2 showed ΔE values clearly distinct from C2. However, the similarity in ΔE values for T1 and T2 indicates that *E. purpurea* extract contributed to color development comparable to that of nitrite. These effects are visually supported by the appearance of the final products, as shown in Fig. 7.

### 3.7 Antimicrobial effect of Echinacea supplementation in fermented sausage

Microbiological evaluation of ripened sausages is crucial for verifying hygienic quality and ensuring consumer safety (Aminzare et al., 2018). As shown in Table S3, no coliform bacteria, *Clostridium botulinum, Salmonella* spp., or *E. coli* were detected in any of the samples throughout the ripening period.

According to the microbiological criteria established in European Commission Regulation No. 2073/2005 (2006), all dry-fermented sausage (DFS) samples met the required safety standards and were considered microbiologically safe for consumption. The fermentation process proved effective in controlling microbial growth, reinforcing its role as a natural preservation method in DFS production.

These findings are consistent with previous studies. Coloretti et al. (2014) reported similar reductions in microbial counts during sausage fermentation, while Pennisi et al. (2020) also observed the absence of *Salmonella* spp. in ripened products. Carvalho et al. (2017) emphasized the significance of total coliforms as indicators of hygienic processing, and Wang et al. (2021) highlighted the rapid inhibitory activity of lactic acid bacteria (LAB) against *E. coli* during fermentation.

### 3.8 Sensory evaluation

Table S4 presents the sensory scores for all treatments, covering attributes such as taste, odor, color, texture, and overall acceptability. The negative control group (C1), which contained neither sodium nitrite nor *Echinacea purpurea*, received the lowest ratings, particularly for taste and texture. This decline may be attributed to the absence of stabilizing additives, which are known to support flavor retention and texture stability during storage.

Conversely, the full-nitrite control (C2, 1.35 % sodium nitrite) achieved the highest sensory scores across all evaluated traits. This aligns with previous findings highlighting the role of nitrites in enhancing color, taste, and texture in cured meat products (Sebranek & Bacus, 2007).

Treatments T1 and T2, which combined a reduced nitrite level (0.67 %) with *E. purpurea* extract (0.05 % and 0.1 %, respectively), showed high sensory acceptability. Their scores were comparable to the full-nitrite control, suggesting that the extract contributed positively to maintaining sensory quality, possibly due to its phenolic content and associated antioxidant properties (Dalby-Brown et al., 2005; Oliveira et al., 2022).

Treatments T3 to T5, which used the plant powder instead of the extract, demonstrated acceptable sensory scores, with T4 receiving slightly better texture ratings. In contrast, T6 (0.2 % powder) showed the lowest taste and texture scores among the treated groups. This may be linked to the higher concentration of bitter or astringent compounds in the powder at this level (Barsett et al., 2012).

Overall, the treatment T2 containing 0.1 % *E. purpurea* extract and 0.67 % sodium nitrite was identified as the most favorable, offering a balanced sensory profile while reducing the reliance on synthetic additives.

## 4 Conclusion

The partial substitution of sodium nitrite with *E. purpurea* extract and powder significantly improved the overall quality of dry-fermented duck sausages. These natural additives enhanced oxidative stability, as evidenced by the marked reductions in TBA and peroxide values, while also supporting favorable moisture retention and protein content. Improvements in color attributes particularly increased redness further contributed to enhanced visual appeal. Among the tested formulations, treatments T2 (0.1 % extract) and T3 (0.2 % extract) achieved the best balance between physicochemical stability and color development. These findings support the use of *E. purpurea* as a viable natural additive for partial replacement of sodium nitrite, promoting the development of cleaner label fermented meat products without compromising safety or quality.

## CRediT authorship contribution statement

**Osama I.A. Soltan:** Writing – review & editing, Writing – original draft, Visualization, Validation, Supervision, Software, Project administration, Methodology, Investigation, Funding acquisition, Formal analysis, Data curation, Conceptualization. **Hanaa S.S. Gazwi:** Writing – review & editing, Writing – original draft, Visualization, Software, Project administration, Methodology, Formal analysis, Data curation, Conceptualization. **Galiya R. Yusupova:** Writing – review & editing, Writing – original draft, Software, Resources, Project administration, Methodology, Investigation, Funding acquisition, Formal analysis, Data curation, Conceptualization. **Andrey P. Gerasimov:** Writing – review & editing, Writing – original draft, Project administration, Methodology, Investigation, Funding acquisition, Formal analysis, Data curation, Conceptualization. **Awatif M. Almehmadi:** Writing – review & editing, Writing – original draft, Software, Project administration, Funding acquisition. **Reem M.E. Magdy:** Writing – review & editing, Writing – original draft, Visualization, Validation, Supervision, Software, Resources, Project administration, Methodology, Investigation, Funding acquisition, Formal analysis, Data curation, Conceptualization.

## Declaration of competing interest

The authors declare that they have no known competing financial interests or personal relationships that could have appeared to influence the work reported in this paper.

## Data Availability

No data was used for the research described in the article.

## Appendix A Supplementary data

Supplementary material Image 1
