# Supplementary material for: Effect of Echinacea purpurea extract and powder with reduced sodium nitrite on quality, oxidative stability, and microbial safety of dry-fermented duck sausage
Source: Food Chem X. 2025 Jul 10;29:102774. doi: 10.1016/j.fochx.2025.102774 (PMC12281069; doi:10.1016/j.fochx.2025.102774)
Supplement: Supplementary file 1 — Supplementary material [file mmc1.docx]

The fermentation process was carried out at a temperature 10 °C and a relative humidity of 94%.

Drying was carried out in a chamber specifically designed for product dehydration, at a temperature of 11 °C and a relative humidity of 78–80%.


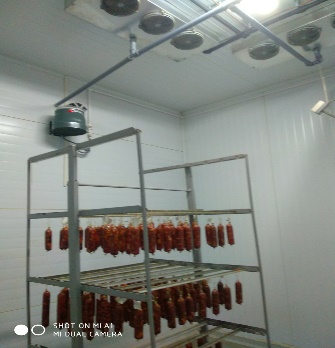

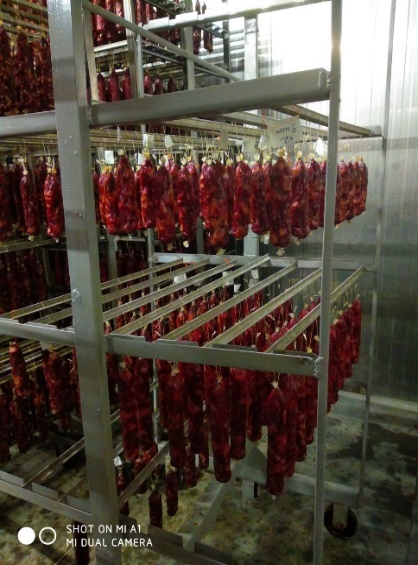

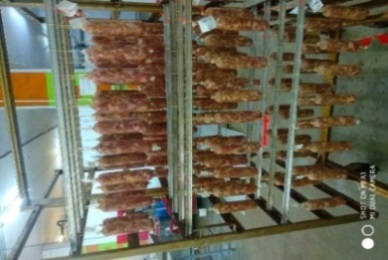


The minced meat of each treatment group was individually mixed in a meat mixer for 15 minutes with salt containing either the full dose of sodium nitrite or a 50% reduced level combined with *Echinacea purpurea* extract or powder. After the initial mixing, duck fat and the remaining ingredients were added and mixed briefly to ensure uniform distribution.

The DFS mixture was stuffed into permeable synthetic casings (Atlantis-Pak Co., Russia) using a vacuum sausage filler, then clipped and looped for hanging during fermentation and drying.


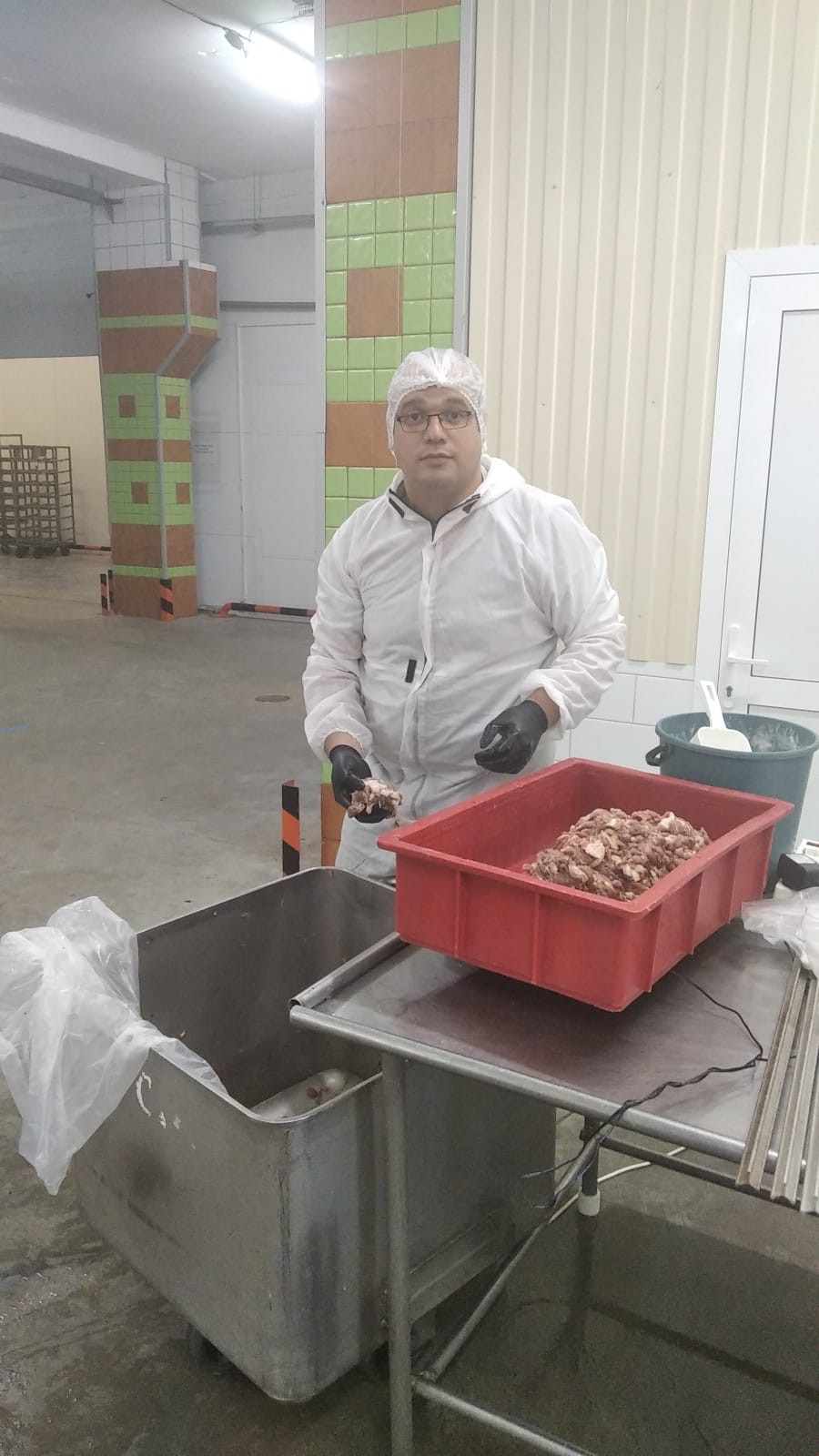

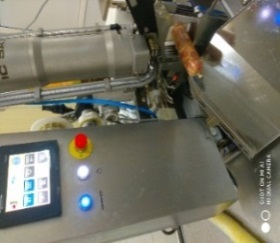


The finished sausages were packaged in vacuum shrink bags, followed by the application of all required labeling and product information.


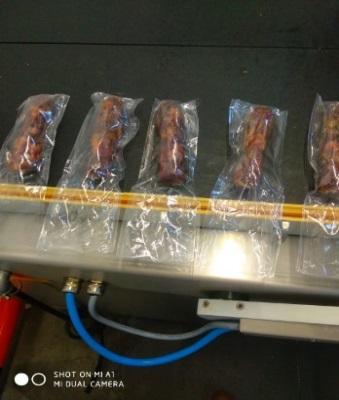


**Fig.S1 Preparation of DFS**

**Table S1 - Recipe for dry- fermented sausage**

| **Sample Materials** | **Amount (Raw materials, kg per 100 kg)** |
| --- | --- |
| Duck breast fillet with skin | 60 |
| Duck fat | 40 |
| Spices and materials, g per 1 kg of unsalted raw materials | |
| Food salt | 1.35 |
| Curing salt (NaNO_2_ = 0.6 %) | C1: Negative control with no curing salt or *Echinacea purpurea* (0 mg/kg NaNO₂)  C2: Positive control with 1.35% curing salt (equivalent to 81 mg NaNO₂/kg meat)  T1- T6: All treatments received 0.67% curing salt (equivalent to 40.2 mg NaNO₂/kg meat), combined with varying concentrations of *Echinacea purpurea* extract or powder |
| Sugar | 0.80 |
| Black pepper powder | 0.20 |
| Dried garlic powder | 0.05 |
| Nutmeg powder | 0.05 |
| Starter cultures "Biobak P"  1.*Lactobacillus sakei:* > 45 x 10^9^ CFU/g  2. *Staphylococcus xylosus*: > 1,0 x 10^10^   CFU/g | 0.05 |
| Echinacea extract or powder | T1 :0.05% Echinacea extract  T2 :0.1% Echinacea extract  T3 :0.2% Echinacea extract  T4 :0.05% Echinacea powder  T5 :0.1% Echinacea powder  T6 :0.2% Echinacea powder |


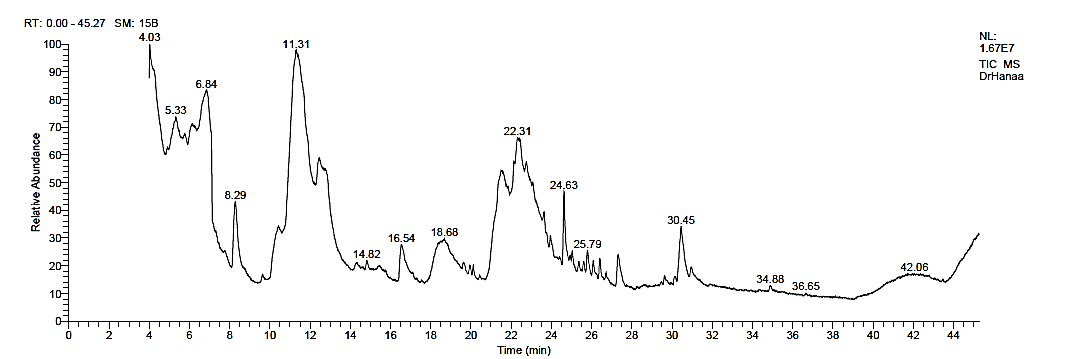


**Figure S2. GC–MS Chromatogram Illustrating Chemical Composition of *Echinacea purpurea***

**Table S2 Phytochemical Constituents Identified in *Echinacea purpurea* Extract by GC-MS**

| **Peak Area %** | **Molecular Weight** | **Molecular Formula** | **Name of the compound** | **RT** | **No.** |
| --- | --- | --- | --- | --- | --- |
| 1.21 | 103 | C₅H₅D₃O₂ | 3,4,4-D₃-trans-3,5-Dihydroxy-cyclopentene | 5.25 | 1 |
| 1.38 | 496 | C₂₀H₃₂O₁₀S₂ | D-Fructose, diethyl mercaptal, pentaacetate | 5.33 | 2 |
| 1.30 | 166 | C₆H₁₄O₅ | 1-Deoxy-D-mannitol | 6.12 | 3 |
| 14.79 | 256 | C₁₆H₃₂O₂ | n-Hexadecanoic acid | 6.93 | 5 |
| 4.84 | 144 | C₆H₈O₄ | 4H-Pyran-4-one, 2,3-dihydro-3,5-dihydroxy-6-methyl- | 8.28 | 6 |
| 1.58 | 287 | C18H30O2 | 10-Heptadecen-8-ynoic Acid, Methyl Ester, (E)- | 10.31 | 7 |
| 1.19 | 184 | C₁₂H₂₄O | 2-Dodecanone | 10.39 | 8 |
| 22.14 | 280 | C₁₈H₃₂O₂ | 9,12-Octadecadienoic acid (Z,Z)- | 11.29 | 9 |
| 2.35 | 158 | C₈H₁₄O₃ | Octanoic acid, 7-oxo- | 12.49 | 10 |
| 2.37 | 130 | C₇H₁₄O₂ | Acetic acid, pentyl ester | 12.74 | 11 |
| 2.39 | 116 | C₆H₁₂O₂ | 1-Butanol, 3-methyl-, formate | 12.80 | 12 |
| 2.81 | 504 | C18H32O16 | Melezitose | 16.53 | 13 |
| 2.29 | 186 | C11H22O2 | Decanoic acid, 3-methyl- | 18.37 | 14 |
| 1.22 | 292 | C19H32O2 | 6,9,12-Octadecatrienoic Acid, Methyl Ester | 18.48 | 15 |
| 1.49 | 278 | C₁₈H₃₀O₂ | 13,16-Octadecadienoic acid, methyl ester | 18.68 | 16 |
| 2.20 | 210 | C₇H₁₄O₇ | d-Glycero-d-ido-heptose | 21.21 | 17 |
| 3.04 | 294 | C₁₉H₃₄O₂ | Methyl linoleate | 21.37 | 18 |
| 5.53 | 302 | C20H30O2 | cis-5,8,11,14,17-Eicosapentaenoic acid | 21.48 | 19 |
| 2.04 | 170 | C₇H₆O₅ | 3,4,5-Trihydroxybenzoic acid | 22.30 | 20 |
| 2.48 | 279 | C10H17NO6S | Desulphosinigrin | 22.38 | 21 |
| 3.31 | 220 | C₁₅H₂₄O | Aromadendrene oxide-(2) | 24.63 | 22 |
| 0.75 | 298 | C₁₉H₃₈O₂ | Octadecanoic acid, methyl ester | 25.03 | 23 |
| 1.06 | 436 | C₂₅H₄₀O₆ | 9,12,15-Octadecatrienoic acid, 2,3-bis(acetyloxy)propyl ester | 25.79 | 25 |
| 0.59 | 410 | C₃₀H₅₀ | Squalene | 26.08 | 26 |
| 0.71 | 296 | C₁₉H₃₆O₂ | 9-Octadecenoic acid, methyl ester | 26.41 | 27 |
| 2.28 | 282 | C₁₈H₃₄O₂ | Oleic Acid | 27.31 | 28 |
| 4.29 | 282 | C18H34O2 | 9-Octadecenoic Acid (Z)- | 30.45 | 29 |

| 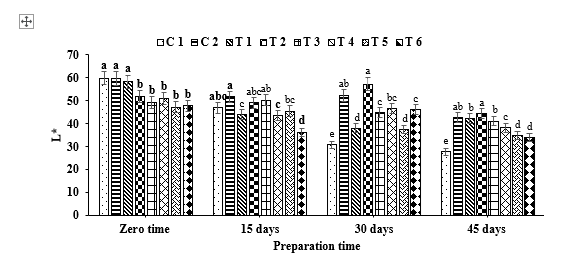 |
| --- |
| 1. **L*** |
| 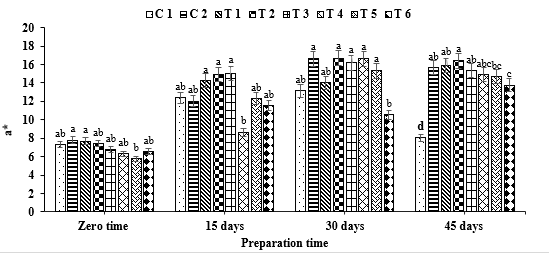 |
| **(b) a*** |
| 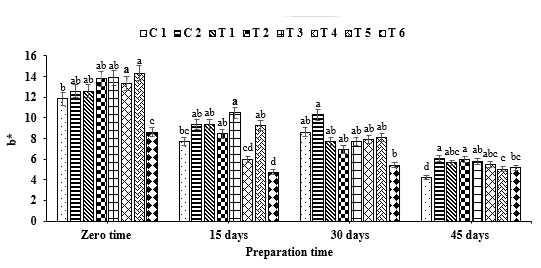 |
| **(c) b*** |
| 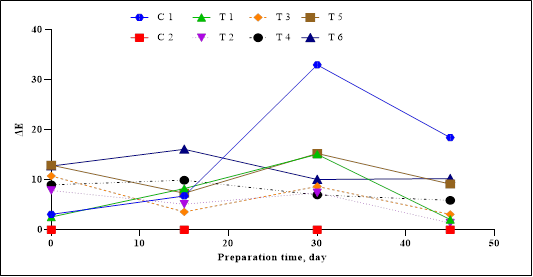 |
| (d) **ΔE** |

**Fig. S3. Changes in color parameters of fermented sausage during preparation: a) Effect of L (lightness), b) Effect of a (redness), c) Effect of b (yellowness), d) Total color difference (ΔE) values.** C1: Negative control with no curing salt or *E. purpurea* (0 mg/kg NaNO₂). C2: Positive control containing 1.35% curing salt (equivalent to 81 mg NaNO₂/kg meat). T1–T3: Treated with 0.05%, 0.1%, and 0.2% *E. purpurea* extract, respectively, each combined with 0.67% curing salt (equivalent to 40.2 mg NaNO₂/kg meat). T4–T6: Treated with 0.05%, 0.1%, and 0.2% *E. purpurea* powder, respectively, each combined with 0.67% curing salt (equivalent to 40.2 mg NaNO₂/kg meat).

**Table S3: microbiological quality of fermented sausage**

|  | **C 1** | **C 2** | **T 1** | **T 2** | **T 3** | **T 4** | **T 5** | **T 6** |
| --- | --- | --- | --- | --- | --- | --- | --- | --- |
| *Coliform bacteria* | n.d. | n.d. | n.d. | n.d. | n.d. | n.d. | n.d. | n.d. |
| *Staphylococcus aureus* | n.d. | n.d. | n.d. | n.d. | n.d. | n.d. | n.d. | n.d. |
| *Listeria monocytogenes* | n.d. | n.d. | n.d. | n.d. | n.d. | n.d. | n.d. | n.d. |
| *Salmonella* | n.d. | n.d. | n.d. | n.d. | n.d. | n.d. | n.d. | n.d. |
| *E. coli* | n.d. | n.d. | n.d. | n.d. | n.d. | n.d. | n.d. | n.d. |
| *Clostridium botulinum* | n.d. | n.d. | n.d. | n.d. | n.d. | n.d. | n.d. | n.d. |

**Table S4. Sensory scores of DFS samples at the end of fermentation**

|  | Taste | Odor | Color | Texture | Overall acceptability |
| --- | --- | --- | --- | --- | --- |
| **C1** | 7.5c±1.50 | 7.6 b±1.27 | 7.4 c±1.35 | 7.4 c±0.69 | 7.6c ±0.84 |
| **C2** | 8.4a ±0.84 | 8.4 a ±0.52 | 8.6 a ±0.69 | 8.5 a ±0.52 | 8.5 a ±0.53 |
| **T1** | 8.3 a±0.95 | 7.8 ab±1.03 | 8.00ab ±0.94 | 7.9 b±0.74 | 8.1 ab ±1.10 |
| **T2** | 8.3 a±0.82 | 8.1 ab ±0.74 | 8.0 ab ±0.81 | 7.9b ±1.19 | 8.0 ab ±1.15 |
| **T3** | 7.8 b±1.22 | 7.7 b±0.95 | 8.0 ab ±0.67 | 8.0 b±0.94 | 7.9 ab ±0.56 |
| **T4** | 8.1 ab±0.99 | 7.6b ±1.42 | 8.1 ab ±0.99 | 8.2 ab ±0.63 | 8.2 ab ±0.92 |
| **T5** | 7.9b ±0.74 | 8.00 ab ±0.94 | 8.00 ab ±1.05 | 7.8b ±1.23 | 7.8 bc±0.79 |
| **T6** | 7.3c ±1.77 | 7.7 b±1.64 | 7.80bc ±0.92 | 7.50c ±0.85 | 8.00ab ±0.67 |

Means within the same column not sharing the same superscript letter (a–c) are significantly different at *P* ≤ 0.05 . C1: Negative control with no curing salt or *E. purpurea* (0 mg/kg NaNO₂). C2: Positive control containing 1.35% curing salt (equivalent to 81 mg NaNO₂/kg meat). T1–T3: Treated with 0.05%, 0.1%, and 0.2% *E. purpurea* extract, respectively, each combined with 0.67% curing salt (equivalent to 40.2 mg NaNO₂/kg meat). T4–T6: Treated with 0.05%, 0.1%, and 0.2% *E. purpurea* powder, respectively, each combined with 0.67% curing salt (equivalent to 40.2 mg NaNO₂/kg meat).
